# Inverse Vulcanization Through Epoxide Chemistry: A Low Temperature Non‐Olefin Route to Sulfur‐Rich Polymer Networks

**DOI:** 10.1002/anie.7470442

**Published:** 2026-06-11

**Authors:** Pan Yang, Youfu Wang, Xinyuan Zhu, Jinhong Jia, Xiaofeng Wu, Tom Hasell

**Affiliations:** ^1^ Department of Chemistry University of Liverpool Liverpool UK; ^2^ Stephenson Institute for Renewable Energy University of Liverpool Liverpool UK; ^3^ School of Chemistry and Chemical Engineering State Key Laboratory of Polyolefins and Catalysis Shanghai Jiao Tong University Shanghai P. R. China; ^4^ State Key Laboratory of High‐Efficiency Coal Utilization and Green Chemical Engineering School of Chemistry and Chemical Engineering Ningxia University Yinchuan Ningxia P. R. China

**Keywords:** epoxide chemistry, inverse vulcanization, recyclable, sulfur polymer, sustainable materials

## Abstract

Inverse vulcanization represents an effective strategy for transforming surplus elemental sulfur into value‐added polymeric materials; however, current approaches are largely restricted to olefin‐based monomers and rely on high‐temperature radical processes. Here, we establish an epoxide‐enabled inverse vulcanization platform that expands sulfur‐rich polymer formation beyond olefin chemistry under solvent‐free, base‐catalyzed conditions. Mechanistic studies confirm a nucleophilic ring‐opening pathway in which sulfur is incorporated into the polymer backbone when catalyzed by base catalyst. By integrating bio‐based epoxidized vegetable oils with elemental sulfur, sulfur‐rich networks are constructed through a catalytically tunable ring‐opening pathway, enabling controllable network formation. The resulting materials maintain high sulfur content while exhibiting tunable mechanical properties, shape memory behavior, and strong adhesion on stainless steel, with lap shear strengths adjustable up to 10 MPa. The combination of mild processing conditions, renewable monomer feedstocks, and robust structural performance demonstrates a controllable and energy‐efficient route for advancing inverse vulcanization toward sustainable adhesive and functional material applications.

## Graphical abstract

Epoxide monomers undergo base‐catalyzed ring‐opening copolymerization with elemental sulfur to afford sulfur‐rich polymers under mild conditions. The resulting materials exhibit excellent mechanical performance, strong adhesion (up to 10 MPa on stainless steel), and outstanding reprocessability.

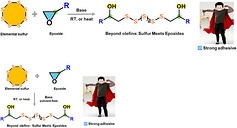

## 1 Introduction

Sulfur is a byproduct of petroleum refining, with an annual production reported to be approximately 70 million tons [1, 2]. Its consumption pathways include, but are not limited to, the production of sulfuric acid [3], fertilizers [4] and rubber vulcanization [5]. However, these processes are unable to consume the surplus sulfur produced annually. Therefore, how to convert excessive sulfur into value‐added materials has attracted significant attention from researchers. In 2013, Pyun and co‐workers [6] reported the pioneering synthesis of sulfur‐rich polymers via a high‐temperature radical addition reaction between elemental sulfur and olefins. This process—inverse vulcanization (IV)‐ provides a direct route to use S_8_ as a monomer for fabricating high‐sulfur‐content polymers. Inverse vulcanized polymers exhibit immense potential across various applications, including high‐refractive‐index materials [7, 8, 9, 10], lithium‐sulfur batteries [11, 12, 13], antimicrobial agents [14, 15, 16, 17], heavy metal adsorbents for wastewater treatment [18, 19, 20, 21], adhesives [22, 23, 24, 25], and high‐performance reprocessable vitrimers [26, 27, 28]. In recent years, IV has garnered widespread attention due to its ability to produce chemically stable and reprocessable sulfur‐rich materials.

Since the first report of radical‐mediated IV in 2013 [6], a significant number of related radical polymerization products have been documented. However, conventional IV typically relies on high‐temperature activation, as the ring‐opening of S_8_ to form polymeric sulfur occurs at the floor temperature of sulfur, <159 °C [29]. Such conditions not only restrict the use of low‐boiling monomers but also introduce safety and scalability challenges, including autoacceleration and the evolution of toxic hydrogen sulfide (H_2_S) [30]. Considerable efforts have therefore been directed toward lowering the reaction temperature. For example, metal diethyldithiocarbamate (DTC) catalysts enable reactions at 100‐135 °C [31], mechanochemical synthesis [32], and photo‐induced processes [33] allow room‐temperature polymerization. In parallel, nucleophilic activation using N‐ or F‐based catalysts promotes S_8_ ring‐opening via an *S_N_* 2 pathway [34, 35, 36, 37, 38, 39, 40], generating reactive polysulfide anions that add to unsaturated bonds. These strategies permit sulfur‐olefin copolymerization under milder conditions, offering a more energy‐efficient alternative to conventional melt processing. Nevertheless, the scope of such systems remains largely restricted to unsaturated olefins and radical polymerization. The nonpolar nature of these monomers often results in brittle materials with limited processability, thereby constraining the broader application of inverse vulcanized polymers.

To overcome the inherent limitations of conventional IV with unsaturated bonds, exploring alternative chemical pathways holds the potential to unlock new monomer libraries and enhance the application profile of IV. The anionic copolymerization of elemental sulfur with strained heterocycles like episulfide and aziridines has been reported; for instance, episulfide can copolymerize with sulfur under anionic conditions [41, 42, 43, 44], typically catalyzed by thiolate salts like sodium thiophenolate, to yield corresponding polysulfides. Grzegorz Mlostoń et al. [45]. reported a mild anionic copolymerization method in which tetrabutylammonium fluoride (TBAF) catalysis the activation of S_8_ to form nucleophilic sulfur species that react with episulfide to yield sulfur‐rich polymers. Ren et al. [46]. reported that episulfide can be selectively converted into disulfide products under the synergistic catalysis of 7‐methyl‐1,5,7‐triazabicyclo [4.4.0] dec‐5‐ene (MTBD), and [PPN]SbF_6_ ([PPN]^+^ is bis(triphenylphosphine)iminium). In this process, S_8_ (or S_x_) is nucleophilically attacked by MTBD to generate reactive sulfur‐based intermediates, which subsequently attack episulfide to form the corresponding disulfide products. Nonetheless, such reactions generally necessitate the use of solvents, and the monomers themselves are characterized by limited availability and complex synthesis [47, 48], rendering the process uneconomical. Recently, Hadjichristidis and co‐workers reported that designed aziridines can undergo step‐growth polymerization with sulfur [49], catalyzed by organic bases. The organobase enables the formation of polysulfide anions from elemental sulfur, which facilitates nucleophilic addition to the aziridine rings. Following this, they also demonstrated the preparation of high‐performance materials from commercially available aziridines and sulfur through a self‐catalytic route [50]. Aziridine‐based sulfur‐rich polymers [23, 51] have been identified as highly effective adhesives. However, this method remains constrained by its dependence on high‐temperature catalysis and limited monomers. These studies suggest that nucleophilic pathways could provide an alternative to radical‐mediated sulfur chemistry; however, practical and sustainable monomer platforms remain scarce. Thus, transitioning toward more sustainable and energy‐efficient protocols remains a significant challenge.

Driven by the need for more versatile synthesis, we have extended our research scope to epoxy‐based monomers. Epoxide monomers are industrially mature and can be abundantly derived from biomass resources, such as vegetable oils [52, 53, 54, 55]. Epoxides have been incorporated into IV primarily through radical‐mediated pathways, either in binary systems containing C═C double bonds [56, 57, 58, 59, 60] or in multicomponent systems where unsaturated monomers first react with sulfur, followed by secondary epoxy ring‐opening reactions [28, 61, 62]. It has been reported that epoxy compounds can undergo ring‐opening reactions with thiol nucleophiles [63, 64] to construct robust polymer networks, suggesting their sensitivity to nucleophilic attack. A notable advancement was achieved by Zhang and colleagues [65], who used anionic hybrid copolymerization to fabricate sulfur‐acrylate materials at room temperature, later extending this to three‐component systems with epoxides [66]. While this represents a significant step toward ambient‐temperature epoxide incorporation, the mechanism fundamentally relies on acrylate‐mediated activation for the ring‐opening process. Consequently, a direct binary copolymerization between elemental sulfur and epoxides remains unachieved within this framework, as sulfur alone cannot trigger the epoxy ring‐opening under these reported conditions. It has been reported that epoxides can undergo nucleophilic addition with sodium polysulfide in polar solvents [67, 68]. A recent study by Liu and colleagues [69] further demonstrated that elemental sulfur can serve as an intrinsic initiator for the synthesis of sulfur‐containing epoxy resins through anion‐initiated epoxy polymerization. Liu's another work represents the first systematic demonstration that epoxy compounds can serve as crosslinkers in IV systems [70] which is radical‐dominated epoxide IV. The synthesis of high‐sulfur‐content epoxy resins is achieved through a mechanism involving sulfur radical‐induced hydrogen abstraction followed by thiol‐epoxy addition. The reaction relies on the generation of sulfur radicals at a high temperature of 180 °C. Despite these foundational studies, previous studies have established the feasibility of incorporating epoxides into sulfur‐based polymer systems; however, these strategies remain confined to either high‐temperature radical chemistry or initiator‐level sulfur incorporation. A practical, controllable, and energy‐efficient sulfur‐epoxide platform in which sulfur functions as a structural component in sulfur‐rich networks has yet to be systematically realized. Addressing this gap is essential for expanding IV beyond its conventional radical paradigm and for unlocking sustainable epoxide feedstocks for advanced functional materials. Moreover, epoxy monomers not only provide a robust mechanical foundation for the materials but also generate a polar microenvironment upon ring‐opening, thereby elevating the functional profile of the sulfur‐rich polymers. This functionality is complemented by the intrinsic reversibility of polysulfide chains, which grants the materials advantageous reprocessability.

**FIGURE 1:**
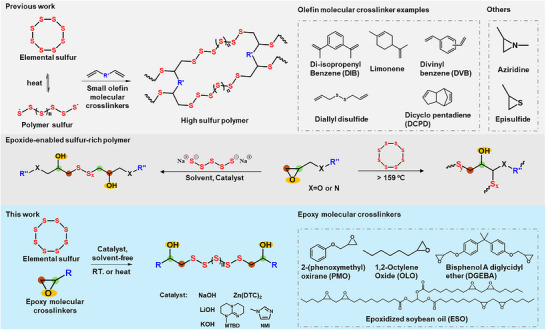
Comparison between previous inverse vulcanization and the catalytic epoxides‐based sulfur‐rich polymer developed in this work. Previous work: Inverse vulcanization typically relies on the thermal or photo activation of elemental sulfur into sulfur radicals, which subsequently react with olefinic crosslinkers or catalyst‐induced nucleophilic attack with other small rings (aziridine, episulfide). This work: A facile base‐catalyzed protocol for the direct copolymerization of elemental sulfur and epoxy molecular crosslinkers (e.g., PMO, OLO, DGEBA, and ESO) under mild conditions (RT or heating). This approach yields sulfur‐rich polymers with hydroxyl groups and high structural diversity.

Herein, we demonstrate that epoxides can serve as a viable monomer platform for IV under mild base‐catalyzed conditions, effectively decoupling sulfur‐epoxide network formation from conventional high‐temperature radical pathways (Figure 1). Through a solvent‐free strategy operable at temperatures as low as ambient conditions, we establish an epoxide‐enabled IV platform that enables controllable sulfur‐rich network construction. By systematically evaluating catalytic systems, we identify both sodium hydroxide (NaOH) and 7‐methyl‐1,5,7‐triazabicyclo [4.4.0] dec‐5‐ene (MTBD) as effective catalysts for promoting sulfur incorporation and regulating network formation. Mechanistic insights derived from model reactions, supported by nuclear magnetic resonance (NMR) and electrospray ionization mass spectroscopy (ESI‐MS) analyses, substantiate the proposed sulfur‐epoxide integration pathway is nucleophilic ring‐opening of S_8_ followed by epoxy chain propagation. The methodology exhibits broad compatibility with aromatic and aliphatic epoxides, underscoring its generality. Notably, adjusting ratio of bio‐based epoxidized vegetable oils and commercial monomer bisphenol A diglycidyl ether (DGEBA) can afford sulfur‐rich networks with tunable mechanical properties, pronounced shape memory behavior, and strong adhesive performance. Overall, this work advances IV beyond its radical‐dominated paradigm and establishes a controllable, energy‐efficient route toward functional sulfur‐rich materials derived from sustainable feedstocks. More broadly, this work expands the monomer scope and mechanistic diversity of IV while mitigating dangerous autoacceleration and toxic hydrogen sulfide production during radical polymerization. It further establishes a foundation for the design and scalable production of sulfur‐based networks under mild and controllable conditions.

## 2 Results and Discussion

Although reactions between epoxides and sulfur have been reported, solvent‐free synthesis has not yet been explored. The development of a green synthetic strategy is of great importance for processable epoxides. To better investigate the reaction between epoxides and sulfur, 2‐(phenoxymethyl) oxirane (PMO) was selected as a model compound for the study. Initially, the reaction between PMO and sulfur was examined under catalyst‐free and solvent‐free conditions. As illustrated in Figure S1, clear phase separation was observed in the PMO‐sulfur mixture after 24 h of reaction. The ^1^H NMR spectrum (Figure S2) revealed no noticeable changes in PMO after the reaction. As an economical and commonly used inorganic compound, NaOH was employed to investigate its catalytic effect on the reaction between PMO and S_8_. The reaction was carried out at 140 °C which is below the floor temperature of sulfur. Sulfur and PMO were first added, and after S_8_ was liquified, a catalytic amount of NaOH (3 wt.% relative to sulfur) was introduced. We found that the reaction system immediately turned dark brown after the addition of NaOH. Within 10 min, the magnetic stirrer stopped rotating due to the increased resistance of the reaction mixture, as shown in Figure S1c and the obtained product was named PMO‐S. As a control, the reaction of PMO under the same catalytic conditions was also carried out and the resulting product was named Poly‐PMO. The structure of the products was subsequently confirmed by NMR and mass spectrometry analyses (Figures S3–S6). As shown in Figure S3, after the reaction between PMO and S_8_, the spectrum becomes significantly broadened, which is consistent with the expected chemical shift distribution caused by oligomerisation. The disappearance of the peaks at 2.7 and 3.2 ppm, attributed to the epoxy three‐membered ring, indicates the complete conversion of PMO. Furthermore, a downfield shift from 3.3 to 4.4 ppm is detected, implying the formation of strongly electron‐withdrawing groups during the reaction. ^13^C NMR, DEPT‐135, and two‐dimensional NMR spectra were employed to further elucidate the structure of the product. The tertiary carbon resonance at 51 ppm shifts to 68 ppm after the reaction, which is consistent with the formation of strongly electron‐withdrawing groups observed in the ^1^H NMR spectrum. Given the presence of oxygen atoms in the epoxy groups, it is proposed that the epoxy rings undergo ring‐opening during the reaction, resulting in the formation of hydroxyl (OH) groups. Combined analysis of the NMR and 2D NMR spectra reveals that the product consists of two PMO units linked by a polysulfide chain via ring‐opened epoxy groups, with concurrent formation of OH groups. To further confirm our proposed hypothesis, ESI‐MS analysis of PMO‐S was carried out (Figure S7). The results are consistent with our assumption, indicating the presence of well‐defined polysulfide chains composed of 2‐6 sulfur atoms in the product. Meanwhile, the self‐polymerization product of PMO (Poly‐PMO) obtained under the same synthetic conditions was also examined (Figures S8‐S11). The ^1^H NMR spectrum exhibits pronounced signal broadening after the reaction, which can be attributed to the inhomogeneous broadening‐induced chemical shift distribution commonly observed in polymeric materials. The ESI‐MS spectrum exhibits a series of well‐defined peaks with regular mass intervals. The detected ions are assigned to sodium adducts of nPMO species with terminal hydroxyl or phenoxy groups, providing strong evidence for the formation of self‐polymerization PMO product. In contrast, these well‐defined and regularly spaced peaks were not observed in the copolymerization product of PMO and S_8_. This result indicates that, in the presence of S_8_ and NaOH, PMO preferentially reacts with S_8_ to form sulfur‐containing compounds. In the infrared spectrum (Figure S12), the characteristic epoxy peaks at 917 cm^−1^ disappeared, accompanied by the appearance of an ‐OH stretching band at 3435 cm^−1^. Meanwhile, characteristic absorption peaks corresponding to C─S and S─S bonds were observed at 644 and 470 cm^−1^, respectively. These results are consistent with the NMR (Figure S13) and MS analyses (Figure S7).

The successful reaction of PMO with S_8_ under NaOH conditions represents a noteworthy and unexpected discovery. This observation reveals that basic catalysts can facilitate C─S bond formation through the reaction between S_8_ and epoxy functionalities. Accordingly, we conducted a systematic investigation into the effects of various basic catalysts on the reaction efficiency between PMO and S_8_. A series of inorganic bases, including potassium hydroxide (KOH), Lithium hydroxide (LiOH) and zinc diethyldithiocarbamate Zn (DTC)_2_, as well as organic bases such as triethylamine, N‐methylimidazole (NMI), and 7‐methyl‐1,5,7‐triazabicyclo (4.4.0) dec‐5‐ene (MTBD), were selected for investigation. As illustrated in Figure S13 and Table S1, the reaction was conducted at 140 °C with a constant catalyst loading of 3 wt.%. The results demonstrate that inorganic bases NaOH, LiOH, as well as organic base MTBD, exhibited the highest catalytic efficiency. Among them, NaOH and MTBD enabled the reaction to reach completion within 10 min, whereas LiOH enabled completion within 60 min. NMI, Zn (DTC)_2_ and KOH also showed favorable catalytic performance. Although apparent phase separation was observed after 4 h, no noticeable phase separation was detected after 24 h, suggesting the complete consumption of PMO and S_8_. In comparison with the other catalysts investigated, triethylamine displayed the lowest catalytic performance. No apparent reaction was observed after 4 h. Even after 24 h, although a black product was formed in the upper layer, distinct phase separation was still observed in the lower layer. Overall, these results highlight the critical role of base strength in governing the efficiency of the PMO‐S_8_ reaction, with NaOH, LiOH, and MTBD emerging as the most effective catalysts for rapid and complete C─S bond formation. Given its low cost, easy accessibility, and excellent catalytic performance, NaOH was selected for further studies.

Catalyst loading was found to be a crucial factor governing the reaction rate. A systematic investigation (Table S2) revealed that at a catalyst loading of 5 wt.%, the reaction proceeded vigorously and was completed within 5 min. When the loading was reduced to 3 wt.%, the reaction remained rapid, reaching completion within 8‐10 min. Further decreasing the catalyst loading to 1‐2 wt.% led to a slower reaction, although full conversion was still achieved within 4 h. Considering both safety concerns related to heat release and overall energy efficiency, a catalyst loading of 3 wt.% was identified as the optimal condition.

**FIGURE 2:**
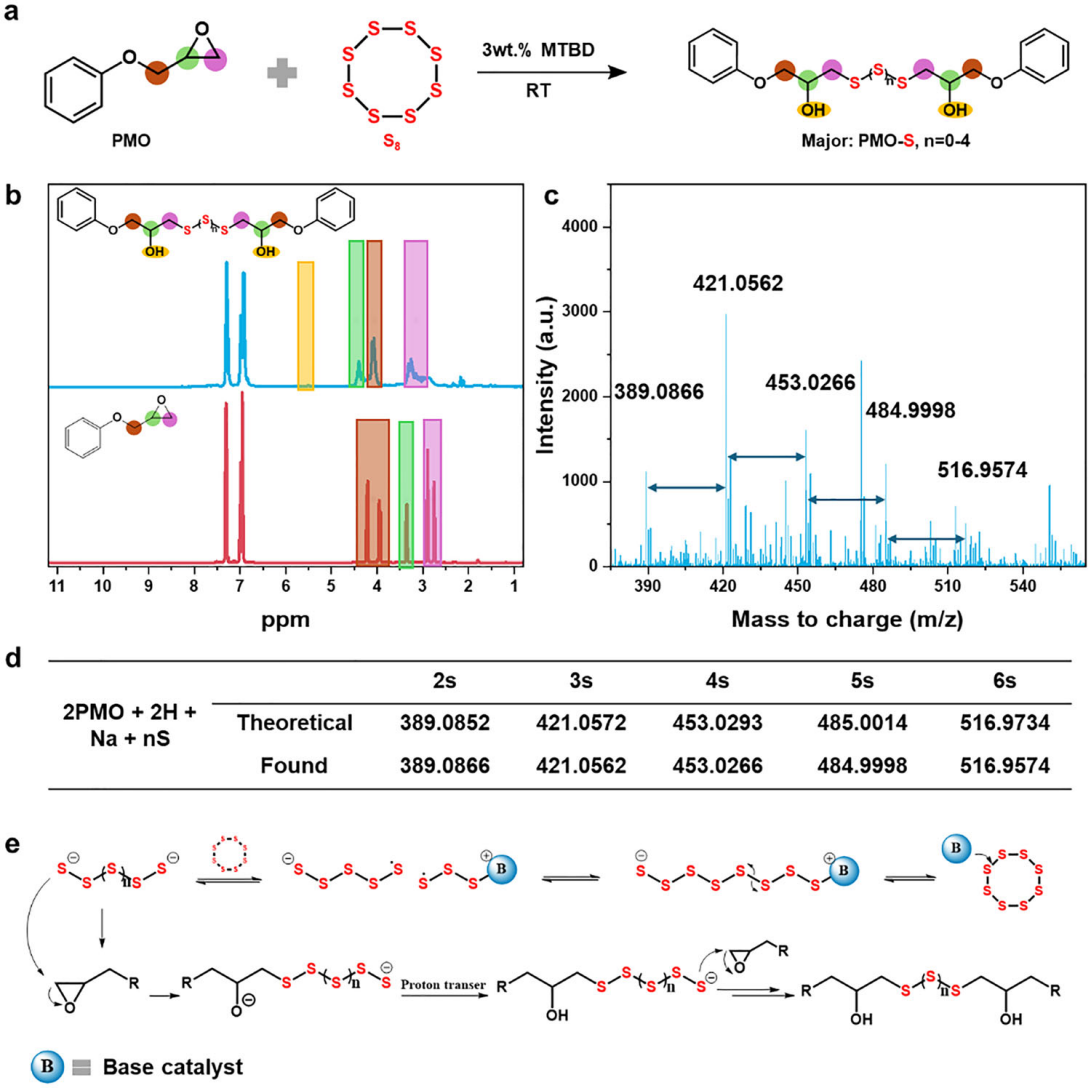
(a) The catalyzed product obtained from the model reaction between PMO and S_8_, (b) ^1^H‐NMR spectra of PMO and PMO‐S. (c‐d) ESI‐MS spectra of PMO‐S. (e) The proposed mechanism of the ring‐opening reaction of PMO with elemental sulfur. Both ionic and radical pathways of the polysulfide chain are illustrated, as both may occur at elevated temperatures, whereas the ionic pathway is expected to dominate at lower temperatures.

The effect of reaction temperature on the NaOH‐catalyzed system was systematically investigated over a range from 140 °C to room temperature. In addition, Fourier transform infrared spectroscopy (FT‐IR) spectroscopy was employed to monitor the reaction, as illustrated in Figures S15–S20 and Table S3, reactions conducted 140 °C rapidly produced a viscous black product within 10 min, with no phase separation observed. At 100 °C (Figures S14,15), the reaction rate decreased but completion was still achieved within 8 h. When the temperature was further lowered to 80 °C (Figures S16,17), the reaction required up to 48 h to reach completion. At 50 °C (Figures S19,20), full conversion took longer than 48 h. By contrast, at room temperature, no significant reaction was detected even after more than 7 days. Accordingly, 50 °C was identified as the minimum effective catalytic temperature for the NaOH‐mediated reaction. The ESI‐MS spectra (Figure S21) of the product obtained at 50 °C were consistent with those of the product formed at 140 °C, indicating identical product structures. Among the inorganic bases examined, LiOH and KOH exhibited relatively slower reaction rates when the temperature was reduced to 100 °C, typically requiring more than 18 h to achieve complete conversion. This behavior is likely associated with the stronger basicity facilitating sulfur activation and accelerating the reaction process [71]. Notably, NaOH displayed the best overall catalytic performance among the inorganic bases investigated, possibly due to its more effective promotion of polysulfide formation and sulfur activation. Among the catalysts examined, MTBD exhibited exceptional catalytic activity, as the reaction was completed within a few seconds after its addition (Figure S22). This prompted us to further evaluate its catalytic performance at room temperature. As shown in the Figures 2a,b, S23,24, the solid in contact with MTBD rapidly changed from yellow to black upon catalyst addition, and the reaction was completed within 48 h. Notably, the ESI‐MS spectra (Figure 2c‐d) of the resulting product were consistent with those obtained from the NaOH‐catalyzed reaction, indicating the formation of identical products.

To better understand the origin of the high catalytic efficiency observed under optimized conditions, we further explored the reaction mechanism. To explore the involvement of radical intermediates, 2,2,6,6‐Tetramethylpiperidinooxy (TEMPO) was introduced into the reaction system as a radical scavenger. As shown in the Figures S25,26, the reaction still proceeded efficiently in the presence of TEMPO. Moreover, the NMR and ESI‐MS spectra of the resulting product were identical to those obtained in the absence of TEMPO, suggesting that radical pathways are not dominant under the present conditions. Nevertheless, we cannot completely exclude the possibility that minor radical processes may occur at elevated temperatures; however, the current experimental evidence does not support a significant contribution from such pathways. Given the basic catalytic environment, we hypothesized that the reaction proceeds via an anionic pathway. To verify this, NaOH and S_8_ were replaced with Na_2_S_5_, and the reaction with PMO was performed at 140 °C. As shown in the Figures S27–30, the reaction reached completion after 72 h. Notably, the NMR, ESI‐MS and FT‐IR spectra of the resulting product were identical to those obtained from the NaOH‐catalyzed system, providing strong evidence that the reaction proceeds through an anion‐induced mechanism. To further investigate the influence of atmospheric moisture on the reaction, we performed the reaction in a glovebox under rigorously anhydrous and oxygen‐free conditions. Interestingly, the reaction proceeded faster under these conditions than under open‐air conditions, with an obvious color change observed after 10 min compared to the open‐air reaction (Figure S31d). The NMR and mass spectrometry results (Figures S31–33) of the products were identical to those obtained in air. Moreover, no gas evolution was observed during the reaction (Figure S31c). We also carried out control experiments with an external water source. The desired products were still obtained smoothly at a slower rate. The behavior is consistent with an anionic ring‐opening mechanism. These results suggest that excess water does not serve as an essential reactant, although trace protic species may participate in the final protonation step. On the basis of the above experimental results, the reaction is proposed to proceed via the anion‐induced mechanism depicted in Figure 2e. Initially, polysulfide anions establish a dynamic equilibrium with elemental sulfur through a scission‐recombination process, mediated by a cationic species (B) to generate active nucleophilic chain ends. These terminal sulfur anions subsequently execute a nucleophilic attack on the epoxide rings, inducing ring‐opening via an *S_N_* 2 pathway to form alkoxide intermediates and new C─S covalent bonds. The resulting alkoxide intermediates are then protonated by trace protic sources, including adventitious moisture [49, 50], monomer impurities, and/or in situ water generated during sulfur activation by NaOH [72], to afford stable secondary hydroxyl groups. Hydrogen abstraction from organic species cannot be fully ruled out; however, the current NMR results do not provide clear evidence that this pathway contributes significantly under the present conditions. Ultimately, this bifunctionalization process yields oligomers capped with hydroxyl moieties at both chain ends and bridged by polysulfide segments.

To investigate the applicability of the proposed mechanism to other epoxide monomers, we selected the fully alkyl‐substituted epoxide, 1,2‐octylene oxide (OLO). The reaction was conducted at 140 °C with 3 wt.% NaOH as the catalyst. A sulfur‐free Poly‐OLO was synthesized under identical conditions as a control group. The resulting products were characterized via NMR, ESI‐MS, and FT‐IR (Figures S34–45). As illustrated in Figures S34,35, the NaOH‐catalyzed reaction reached completion within 30 min, yielding a viscous black solid. Furthermore, the methine proton (CH) signal shifted from 2.6 to 3.9 ppm, which is ascribed to the formation of strong electron‐withdrawing groups. The ^13^C NMR and DEPT‐135 characterization further confirmed the structural transformation; the signals corresponding to the tertiary carbons underwent a significant downfield shift from 52 to 69 ppm upon the formation of the hydroxyl moieties. The ESI‐MS spectra indicate that the product contains 2‐7 sulfur atoms. Notably, unlike Poly‐OLO, which displays a series of well‐defined peak clusters with a uniform interval of 128.12 Da, such characteristic peak clusters are not observed in OLO‐S, suggesting that in the presence of S_8_, OLO preferentially reacts with sulfur rather than undergoing self‐polymerization under NaOH catalysis.

The results obtained from the model compound studies confirm that basic catalysts can efficiently catalyze the anionic ring‐opening copolymerization of epoxy groups with S_8_. Motivated by these findings, we chose commercially available DGEBA, which contains two terminal epoxy groups, as the monomer for the synthesis of linear polymeric materials which is named as DGEBA‐S. As shown in the Figures S46–48, the reaction proceeded rapidly and reached completion within 5 min. The pronounced peak broadening further indicates the formation of polymeric species. FT‐IR analysis provided additional evidence for the structural transformation. After polymerization, a new absorption band at 3368 cm^−1^, corresponding to OH groups, was observed, while the characteristic epoxy ring vibrations at 915 cm^−1^ disappeared. Differential scanning calorimetry (DSC) analysis (Figure S49) revealed that the glass transition temperature (*T*_g_) of the resulting material was 50.4 °C, which is substantially higher than that of Poly‐DGEBA (8.1 °C) prepared under the same conditions. However, during NMR characterization, DGEBA‐S was found to be only partially soluble, contradicting our expectation of forming a purely linear polymer. To further investigate this unexpected behavior, elemental analyses of the chloroform‐soluble and insoluble fractions were conducted, with the results presented in Figures S50, S51 and Table S4. Elemental analysis revealed a significant compositional disparity between the soluble and insoluble fractions of the DGEBA‐S polymer. The soluble part exhibited a markedly lower C/H ratio (9.98) compared to the insoluble part (10.44), which is primarily attributed to its high sulfur content (64.74%) and the enrichment of low‐molecular‐weight oligomers with longer polysulfide chains. In contrast, the insoluble part shows a C/H ratio closer to the theoretical value, indicating the formation of a dense, cross‐linked network where the DGEBA segments are effectively integrated. Such branching or crosslinking could be caused by reaction of epoxy groups with any pendant hydroxyl group on another growing polymer chain (depicted in Figure S51). Furthermore, the significantly lower sulfur content in the insoluble fraction implies that the DGEBA‐derived backbone is preferentially concentrated within the cross‐linked matrix rather than the sulfur‐rich soluble fragments.

Petrochemical products play a vital role as raw materials in the chemical industry and are widely used in industrial production. However, with the increasing consumption of petroleum, global oil prices have remained high, and the depletion of fossil resources has become a growing concern. Under these circumstances, the development of low‐cost, easily accessible, and renewable biomass resources as alternatives to petrochemical feedstocks has become particularly important. Vegetable oils have attracted widespread attention due to their advantages of low cost, ease of cultivation, environmental friendliness, and renewability. The carbon‐carbon double bonds in vegetable oils can be converted into epoxide groups through epoxidation reactions in the presence of catalysts, producing epoxidized vegetable oils. These environmentally friendly bio‐based epoxides have been widely used as additives in various materials and have drawn significant interest from the scientific community. Among them, epoxidized soybean oil (ESO) has the highest production volume and has already been industrialized.

Commercially available ESO with an epoxy value of 6.6 was selected for the synthesis of cross‐linked compounds. As illustrated in Figure S52, ESO does not react with S_8_ in the absence of the catalyst NaOH. However, upon the addition of NaOH, the reaction between ESO and S_8_ yields a black sticky solid. For comparison, the self‐polymerization product of ESO was also prepared. ^1^H NMR spectra (Figures S53,54) reveal that the resonance peaks broaden significantly after the reaction, which is a characteristic signature of polymerization. In the FT‐IR spectra (Figure S55), the characteristic peak at 842 cm^−1^ corresponding to the C─O─C stretching vibration of the epoxy ring disappeared after the reaction. Simultaneously, new absorption peaks emerged at 674 cm^−1^, which can be assigned to the C─S bond, respectively. Furthermore, a strong and broad band appeared at 3433 cm^−1^, characteristic of the stretching vibration of ‐OH groups, indicating the successful ring‐opening of the epoxy groups. Experimental results demonstrate that the synthesized ESO‐S exhibits excellent solubility in chloroform, indicating that a highly cross‐linked network was not formed. This may be attributed to the saponification [73] of the ester bonds in the ESO under alkaline reaction conditions, which subsequently restricted the further expansion of the three‐dimensional cross‐linked framework. The molecular weight distribution was analyzed via Gel Permeation Chromatography (GPC). The GPC results reveal that ESO‐S possesses a broad molecular weight distribution and a high weight‐average molecular weight (Table S5). In contrast, the sulfur‐free Poly‐ESO shows a narrower distribution and significantly lower molecular weight. This comparison provides strong evidence that the self‐polymerization capability of ESO is relatively weak under identical reaction conditions. However, the introduction of sulfur facilitates the reaction with monomers, significantly promoting the formation of high‐molecular‐weight products. Furthermore, the soluble fraction of DGEBA‐S is characterized by a narrow distribution and high molecular weight, suggesting a higher degree of structural regularity or specific reaction selectivity during its polymerization process. As shown in the DSC curves (Figure S56), the *T*_g_ of ESO‐S is ‐13.4 °C which is lower than that of DGEBA‐S. This difference can be attributed to the greater flexibility of the longer aliphatic chains in ESO compared with the shorter and more rigid aromatic structure of DGEBA. The endothermic peak of ESO‐S is located at 18 °C, which can be attributed to glycerol. The result further confirming the successful synthesis of the copolymer derived from ESO and S_8_.

The abundance of dynamic S‐S linkages inherent in inverse vulcanized polymers makes them premier candidates for the fabrication of vitrimers, endowing the materials with reprocessability and self‐healing capabilities. To bridge the gap between laboratory research and industrial implementation, researchers are dedicated to enhancing their mechanical robustness through monomer optimization and network engineering, thereby overcoming the inherent brittleness of traditional high‐sulfur materials. Therefore, we developed a ternary system designated as S_8_‐ESO‐DGEBA (hereafter referred to as SED), to leverage the synergy between the components: DGEBA contributes rigidity and density, and ESO contributes to the network density and elasticity. The synthesis details of SED are documented in the Supporting Information. Three series of SED terpolymers (5–5–x, 5–7.5–x, and 5–10–x; the numbers denote the gram amounts of sulfur, ESO, and DGEBA, respectively) with different weight ratios were prepared for investigation (Table S6).

**FIGURE 3:**
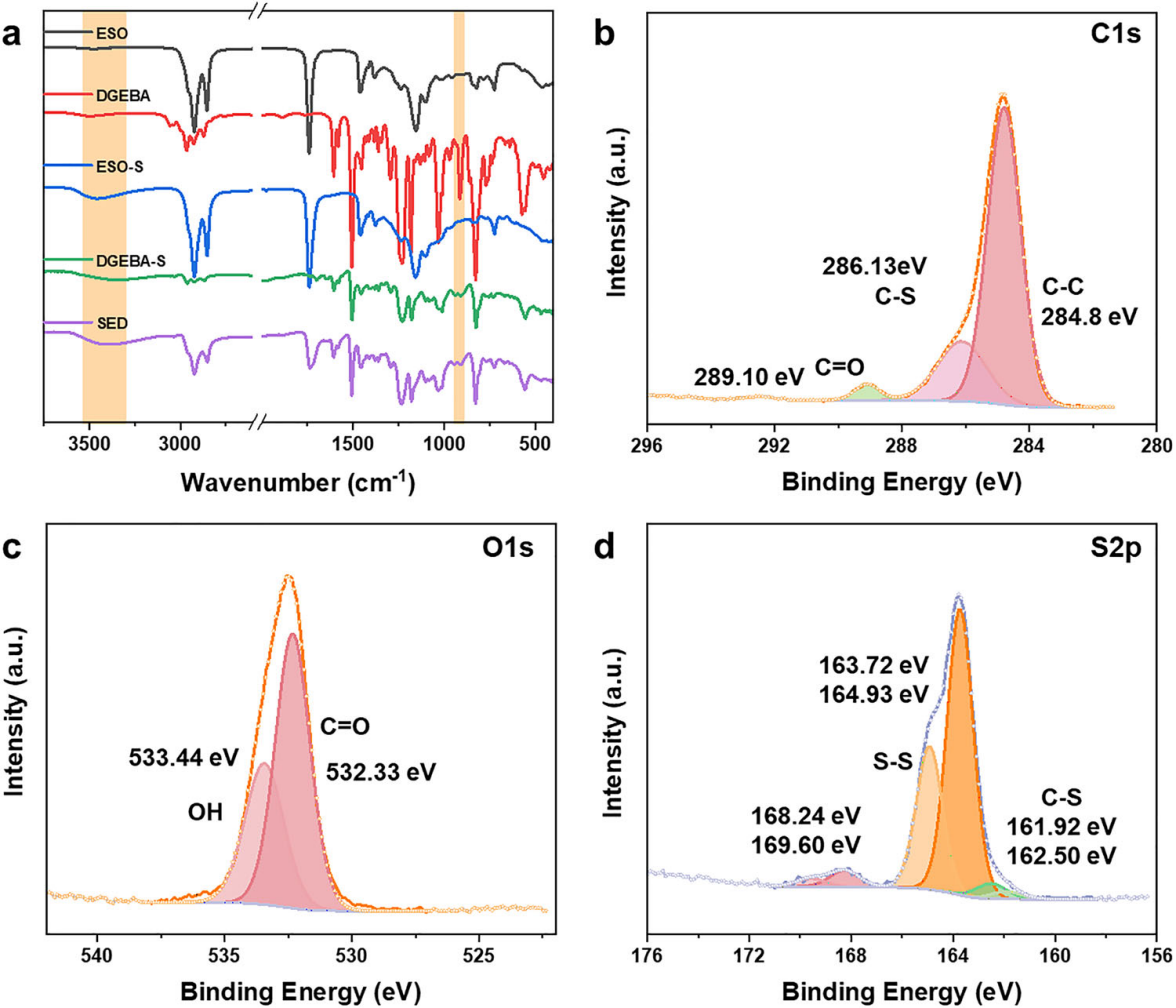
Characterization of polymer SED. (a) FT‐IR plot about SED (purple), (b) C1s XPS spectrum of SED. (c) O1s XPS spectrum of SED. (d) S2p XPS spectrum of SED.

FT‐IR and x‐ray photoelectron spectroscopy (XPS) was employed to further corroborate the chemical structure of the synthesized polymers. As shown in Figure 3a, a characteristic peak belonging to the ‐OH group appeared at 3433 cm^−1^ in the SED product after the reaction. Meanwhile, the characteristic peaks of the epoxy group from DGEBA at 1133 and 970 cm^−1^ disappeared. The XPS survey spectrum (Figures 3b‐d and S57–59) displays only C, S, and O signals, suggesting a robust interaction between the monomers and S_8_. In the high‐resolution C 1s spectrum, the deconvoluted peaks at 284.8 eV and 286.2 eV correspond to C─C and C─S bonds, respectively, while the signal at 289.1 eV is assigned to the C═O bond. The O 1s spectrum reveals a C═O peak at 532.3 eV and a characteristic ‐OH peak at 533.4 eV, confirming the successful ring‐opening of the epoxy groups and the formation of hydroxyl moieties. Regarding the S 2p spectrum confirms S‐S (163.7/164.9 eV) and S‐C (161.9/162.5 eV) linkages. The peaks at 168 and 169 which belong to sulfonic acid group [74, 75]. The simultaneous presence of ‐OH and C‐S functionalities confirms that the S_8_ ring successfully reacted with the electrophilic carbon of the epoxy group, resulting in the desired C–S coupling and hydroxyl generation. Solubility tests (Figure S60, Table S7) showed that the polymer could be completely dissolved in DMF and DMSO. This may be because polysulfide bonds can break in polar solvents [22, 28]. Thermal characterization of the resulting polymers was performed, such as DSC and TGA (Figures S61–64). The results indicate that increasing the content of the rigid component DGEBA increases the *T*_g_, reaching a maximum value at room temperature. Since the glass transition is a characteristic of linear polymer chain segments, an increase in crosslinking degree reduces the intensity of this transition. The thermal degradation behavior of all polymers followed a similar decomposition profile, initiating between 170 °C and 200 °C, with final char yields varying slightly based on composition but generally falling within the 15%–25% range. These thermal behaviors align with previously reported trends observed in inverse vulcanized polymers. Accordingly, the successful preparation of a crosslinked terpolymer was achieved.

**FIGURE 4:**
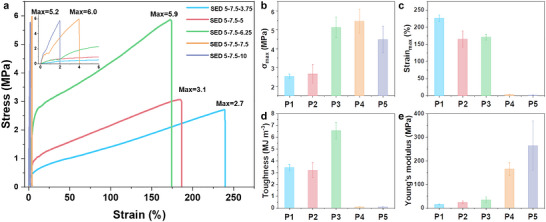
(a) The typical strain‐stress curves of SED sulfur‐polymers. The subpanel shows an expansion of the initial strain section (0‐6 %) of the same graph. A summary of mechanical performances of the S_8_ copolymers. Measurements were carried out in triplicate (*n* = 3); error bars show the standard deviation of the replicate measurements; the error bar centers are the means of the replicate measurements. (b) The stress at break, (c) the strain at break and (d) toughness of crosslinked polymers SED, (e) Young's modulus of crosslinked polymers SED. P1‐P5 correspond to SED 5‐7.5‐X with X = 3.75, 5, 6.25, 7.5, and 10, respectively.

Chemical cross‐linking is widely considered to enhance the mechanical performance of polymers. The mechanical performance of the SED polymers was systematically evaluated through tensile testing, as illustrated in the stress‐strain curves across three distinct series (Figure 4). A consistent trend is observed: the mechanical behavior of the materials undergoes a dramatic transition from a soft, ductile elastomer to stiff, brittle materials as the concentration of DGEBA increases.

In the SED 5‐5‐x series, the polymers exhibit relatively low tensile strengths (0.4‐1.4 MPa) but maintain moderate ductility (Figure S66). However, by decreasing the ration of sulfur in the SED 5‐7.5‐x and SED 5‐10‐x series (Figures 4 and S67), the maximum tensile strength is significantly increased, reaching peaks of over 6.0 MPa. This enhancement is attributed to the increased cross‐linking density. This observation aligns with the material design strategy, where the ESO provides the cross‐linked network, while DGEBA contributes both structural rigidity and additional cross‐linking sites.

Specifically, SED 5‐7.5‐6.25 represents a critical “sweet spot” in formulation, achieving an optimal balance between strength (5.86 MPa, Figure 4b) and flexibility (∼175% strain, Figure 4c). Beyond this threshold, such as in the case of SED 5‐7.5‐10 (Figure 4d,e) or SED 5‐10‐10, the excessive cross‐linking restricts the mobility of the linear polymer chain segments so severely that the material becomes highly brittle, failing at negligible strain levels due to stress concentration. These results demonstrate that the mechanical hierarchy of SED polymers can be precisely tuned by modulating the stoichiometric ratios of the reactants.

The disulfide (S─S) linkage is well‐known for its ability to undergo thermally induced dynamic exchange reactions, a chemistry widely exploited in vitrimeric systems to impart recyclability to otherwise intractable thermosetting resins. Given their sulfur‐chain backbone, the synthesized SED polymers were expected to exhibit dynamic characteristics which is similar with disulfide‐based vitrimers. To verify this, the reprocessability of the representative SED 5‐7.5‐6.25 sample was evaluated through multiple recycling generations (Figures S68,69).

As anticipated, the material maintained substantial mechanical integrity throughout the testing cycles. The pristine sample exhibited a tensile strength of 5.86 MPa, which transitioned to 4.66 MPa (79.5% retention) and 4.20 MPa (71.7% retention) after the first and second recycling cycles, respectively. This moderate decline may be attribute to minor chemical degradation or slight inefficiencies in network reconstruction during re‐molding. Notably, however, a unique recovery trend emerged during the third cycle, where the tensile strength rebounded to 4.55 MPa. This unexpected enhancement points toward a thermal‐induced structural optimization effect. It is hypothesized that repeated high‐temperature processing facilitated the re‐equilibration of the dynamic covalent, resulting in a more homogeneous and robust cross‐linked architecture. The decrease in *T*_g_ (Figure S69) after repeated measurements further suggests that structural rearrangement has occurred. This self‐reinforcing capability, alongside a consistently high strength retention (> 70%), underscores the exceptional structural stability and sustainability of the SED system. This recyclability is crucial for the development of sustainable and eco‐friendly polymer materials and is not possible with currently widely used epoxy resins and composites.

**FIGURE 5:**
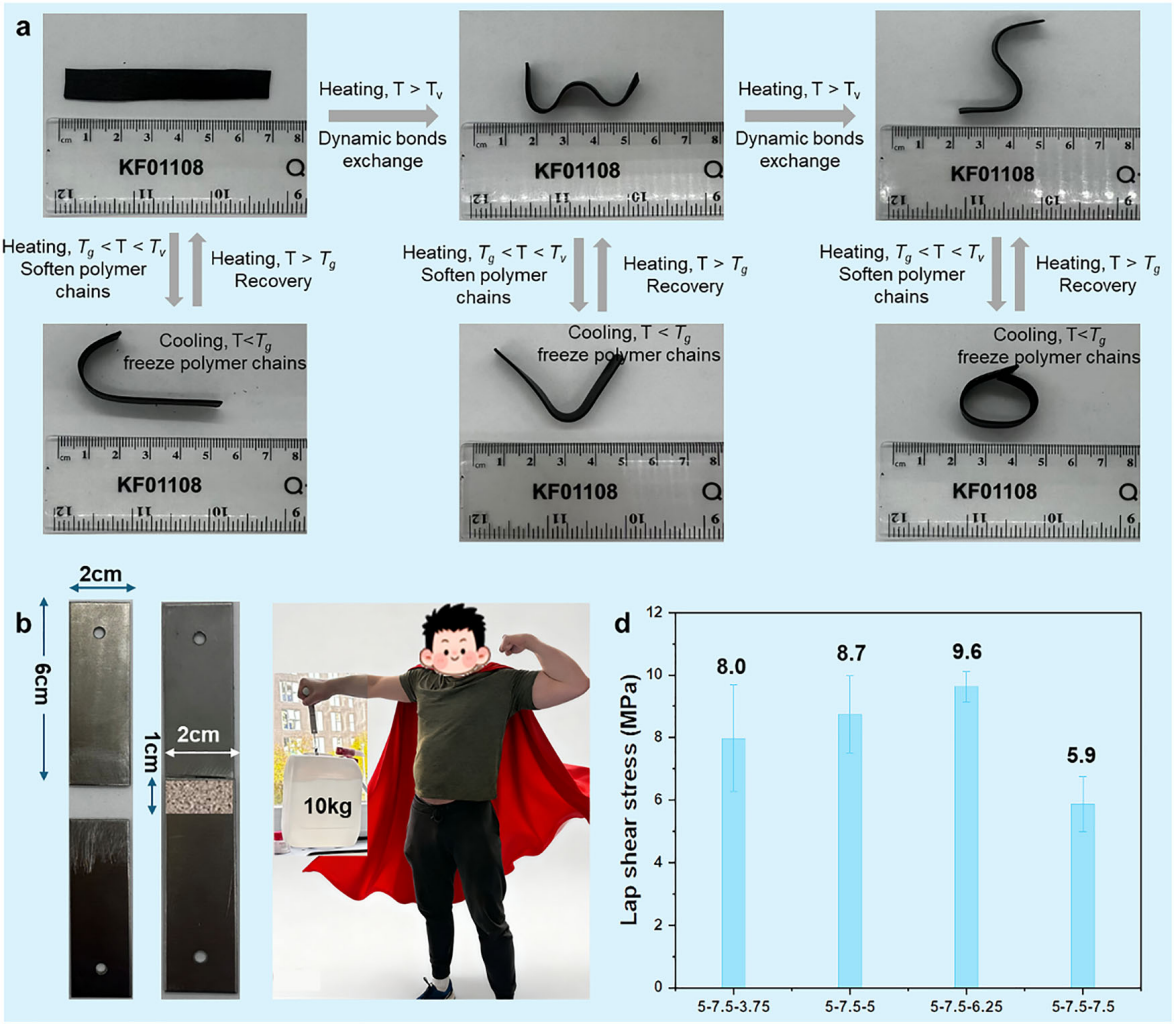
(a) Example of reversible shape memory performance and the cumulative plasticity reprogramming process of crosslinked polymer SED (5‐7.5‐6.25). (b‐c) Image showing two steel substrates bonded with SED (5‐7.5‐6.25) withstanding a force of 10 kg; (d) lap shear strength of different SED cross‐linked polymers with stain‐less steel sheet.

The shape memory behavior of conventional polymers, along with their underlying mechanisms, has been investigated for many years. However, the inherent reversibility of long sulfur chains in IV polymers also imparts significant shape memory effects. In addition to reversible state transitions, the dynamic chemistry of S─S bonds enables topological rearrangement, allowing for the reconfiguration of the polymer's permanent shape‐a characteristic typical of advanced vitrimeric materials. To further investigate the stimulus‐responsive behavior and structural plasticity of the SED polymers, a shape memory and permanent shape reconfiguration experiment was conducted. As demonstrated in Figure 5a, the material exhibits versatile shaping capabilities. The top row of images displays three distinct permanent shapes (straight strip, ‘W’ shape, and ‘S’ shape). Unlike traditional thermosets, these permanent geometries are not fixed during initial curing but are instead reconfigured through thermally induced network rearrangement. By applying heat at elevated temperatures (above the topology freezing transition temperature, Tv), dynamic disulfide exchange allows the cross‐linked network to relax and adapt to new equilibrium states, effectively ‘programming’ a new permanent architecture. The bottom row illustrates the corresponding temporary shapes (curved, ‘V’ shape, and ring) achieved through a standard shape‐memory cycle. Each temporary state was fixed by cooling the material below its *T*_g_, which ‘freezes’ the polymer chain segments in a strained state. Upon reheating, the entropic elasticity of the network drives the material back to its current permanent form with high fidelity. Notably, the SED polymer successfully underwent three successive cycles of permanent reconfiguration and temporary deformation without observable macro‐fracture or loss of structural integrity. These processes are illustrated in two videos provided in the Supporting Information, which correspond to the reversible shape‐memory behavior between shapes (v) and (w), and between shapes (o) and (s), respectively. This dual‐responsive characteristic‐combining reconfigurable plasticity with reversible shape memory‐demonstrates the robust nature of the dynamic sulfur‐based covalent network.

The SED polymer architecture contains abundant hydroxyl moieties. These groups are anticipated to facilitate strong interfacial interactions with polar functional groups on the substrate surface‐via hydrogen bonding and van der Waals forces‐to realize adhesion properties. The adhesive performance of the SED polymers was evaluated using stainless steel substrates through lap shear tests (Figures 5b–d and S70–72). The results indicate that the lap shear strength is highly dependent on the stoichiometric ratio of the components. As the third component increases, the shear stress peaks at 9.6 MPa for the SED 5‐7.5‐6.25 formulation (Figure 5d). This exceptional adhesion is attributed to the synergistic effect of the abundant hydroxyl (‐OH) groups and the flexible sulfur‐rich backbone, which facilitate strong interfacial interactions and effective energy dissipation. However, a further increase in the component ratio (e.g., 5‐7.5‐7.5) leads to a decline in adhesive strength (5.87 MPa), likely due to increased brittleness and stress concentration at the interface. The practical robustness of this SED adhesive is vividly demonstrated in Figure 5c and the video in the Supporting Information, in which it successfully supports a heavy load, showcasing its potential as a high‐performance, sustainable structural adhesive. Meanwhile, the abundant disulfide bonds (S─S bonds) within the SED network enable dynamic exchange characteristics, endowing the material with excellent reusability. The formulation with the superior adhesive performance (SED 5‐7.5‐6.25) was selected to evaluate the recyclability of its adhesion properties. Detailed experimental procedures are provided in the Supporting Information. As illustrated in Figures S73,74, the material maintains robust adhesive strength even after three consecutive bonding‐debonding cycles. The ability of the material to support heavy loads while remaining reconfigurable and recyclable highlights its immense potential for sustainable engineering applications. The developed material was compared with a commercially available two‐component epoxy adhesive and other inverse vulcanized polymers (Figure S75 and Table S8). The results (Figure 5d) show that the maximum lap shear strength of the prepared material reaches 9.6 MPa, which is comparable to that of the commercial counterpart (approx. 11 MPa). Notably, unlike traditional thermosetting epoxy systems, this material exhibits excellent reprocessability, recyclability and debondable. The adhesive performance of the obtained material was further evaluated on various substrates, including glass, wood, and PVC (Figures S76–79). In all cases, the material exhibited strong interfacial adhesion, demonstrating its broad substrate compatibility. Notably, during lap shear testing on glass substrates, fracture of the brittle glass occurred prior to adhesive failure, preventing measurement of the ultimate adhesive strength. This observation suggests that the interfacial adhesion exceeded the mechanical tolerance of the glass substrate itself. The SED polymers likely interact favorably with the polar surface groups of stainless steel (9.6 MPa), wood (5.9 MPa), and glass (≥ 4.2 MPa), resulting in strong interfacial adhesion. In contrast, the relatively nonpolar PVC surface led to weaker adhesion (1.6 MPa), with detachment of the polymer from the substrate observed after testing. These features provide a significant competitive edge in terms of environmental sustainability and practical applications.

## 3 Conclusion

In summary, we have established an epoxide‐enabled inverse vulcanization platform that expands sulfur‐rich polymer formation beyond conventional olefin‐based, high‐temperature radical systems. Through a solvent‐free and base‐catalyzed strategy operable at temperatures down to ambient conditions, sulfur was successfully integrated into epoxy‐derived networks via a controllable ring‐opening pathway. Systematic investigation of catalytic systems revealed that both NaOH and MTBD effectively regulate sulfur incorporation and network formation. The resulting sulfur‐rich networks, particularly those derived from bio‐based epoxidized vegetable oils, exhibit adjustable mechanical properties, pronounced shape memory behavior, and strong adhesive performance with lap shear strengths up to 10 MPa. The synergy between sulfur incorporation and epoxy‐derived polar network formation provides a balanced combination of mechanical robustness and processability, while the intrinsic reversibility of polysulfide linkages contributes to reconfigurability and adaptive material characteristics.

Beyond the material systems discussed above, we decouple sulfur‐epoxy network formation from the high‐temperature radical paradigm by introducing a controllable, energy‐efficient synthetic strategy. This approach suppresses self‐accelerating reactions and toxic H_2_S evolution associated with conventional IV, offering scalability and new opportunities for advancing the field. Future studies may focus on detailed mechanistic elucidation of the catalytic sulfur activation pathway, expansion to other epoxide architectures and multifunctional monomers, and integration with composite or interface‐engineered systems for structural adhesive, recyclable thermoset, and smart material applications. The development of predictive structure‐property relationships and computationally guided materials design may further accelerate the rational design of next‐generation sulfur‐rich polymer networks.

## Author Contributions

**Pan Yang**: conceptualization, methodology, investigation, validation, formal analysis, data curation, visualization, project administration, writing ‐ original draft, writing ‐ review and editing. **Youfu Wang**: investigation, formal analysis. **Xinyuan Zhu**: supervision. **Jinhong Jia**: investigation, formal analysis, conceptualization. **Xiaofeng Wu**: project administration, writing ‐ review and editing, supervision. **Tom Hasell**: supervision, project administration, writing ‐ review and editing, funding acquisition, conceptualization.

## Conflicts of Interest

The authors declare no conflicts of interest.

## Supporting Information

The authors have cited additional references within the Supporting Information.[76–89].

## Supporting information




**Supporting File 1**: anie73141‐sup‐0001‐MovieS1.mp4

**Supporting File 2**: anie73141‐sup‐0002‐MovieS2.mp4

**Supporting File 3**: anie73141‐sup‐0003‐MovieS3.mp4

**Supporting File 4**: anie73141‐sup‐0004‐SuppMat.docx

## Data Availability

All data needed to evaluate the conclusions in the paper are present in the paper and/or the Supplementary Information.
