# Supplementary material for: Inverse Vulcanization Through Epoxide Chemistry: A Low Temperature Non‐Olefin Route to Sulfur‐Rich Polymer Networks
Source: Angew Chem Int Ed Engl. 2026 Jun 11;65(33):e7470442. doi: 10.1002/anie.7470442 (PMC13452515; doi:10.1002/anie.7470442)
Supplement: Supplementary file 1 [file ANIE-65-e7470442-s001.docx]

**Supplementary information**

**Inverse Vulcanization through Epoxide Chemistry: A low-temperature Non-Olefin Route to Sulfur-Rich Polymer Networks**

Pan Yang^a,^^b^*, Youfu Wang^c^, Xinyuan Zhu^c^, Jinhong Jia^d^, Xiaofeng Wu^a^*, Tom Hasell^a,b^*

[a]: Department of Chemistry, University of Liverpool, Liverpool L69 7ZD, United Kingdom

[b]: Stephenson Institute for Renewable Energy, University of Liverpool, Liverpool L69 7ZF, United Kingdom

[c]: School of Chemistry and Chemical Engineering, State Key Laboratory of Polyolefins and Catalysis, Shanghai Jiao Tong University, Shanghai 200240, P. R. China.

[d]: State Key Laboratory of High-Efficiency Coal Utilization and Green Chemical Engineering, School of Chemistry and Chemical Engineering, Ningxia University, Yinchuan, 750021, Ningxia, China

*Corresponding Author

Pan Yang

Xiaofeng Wu

Tom Hasell

ORCID ID: orcid.org/0000-0003-4736-0604

Email: [t.hasell@liverpool.ac.uk](mailto:t.hasell@liverpool.ac.uk)

**Materials**

Sulfur (S_8_, ≥99.5%) was purchased from Brenntag UK & Ireland. 2-(phenoxymethyl) oxirane (PMO), 1,2-octylene oxide (OLO), bisphenol a diglycidyl ether (DGEBA), sodium hydroxide (NaOH), potassium hydroxide (KOH), Lithium hydroxide (LiOH), triethylamine, N-methylimidazole (NMI), 7-methyl-1,5,7-triazabicyclo (4.4.0) dec-5-ene (MTBD), zinc diethyldithiocarbamate (Zn (DTC)_2_) were purchased from Sigma Aldrich. Epoxidized soybean oil (ESO) was purchased from ‎Eastchemlab, epoxy value ≥ 6.6. Epoxy two-component adhesive was purchased from Amazon, sold by HIVETZ. Other solvents were purchased from Fisher Chemical. All chemicals and solvents were used as received without further purification.

**Characterizations**

**Fourier Transform infrared Spectroscopy (FTIR).** FTIR was conducted using a Vertex 70 Fourier Transform Infrared Spectrometer equipped with an attenuated total reflection (ATR) cell with a range of 400-4500 cm-1 for 32 scans under a resolution of 4 cm^-1^.

**Liquid-State Nuclear Magnetic Resonance (NMR).** All liquid-state NMR spectra were recorded at 400 MHz on a Bruker Avance Ⅲ HD spectrometer. Deuterium chloroform-d was used. Chemical shifts are reported in ppm with reference to internal residual protonated species of the deuterated solvents used for ^1^H and ^13^C analysis. ^1^H chemical shift reference to the residual CHCl_3_ (in CDCl_3_) at 7.26 ppm and with ^13^C shift of 77.03 ppm.

**CHNS Elemental Analysis (EA).** EA was performed by Elementar Vario MicroCube.

**Thermogravimetric Analysis (TGA).** TGA was conducted using a TA instruments TGA550 with Rh/Pt wire wound furnace at a heating rate of 10 ℃/min in the range of 25-700 ℃ under nitrogen atmosphere.

**Differential Scanning Calorimetry (DSC).** DSC operated by a TA instruments discovery series DSC 25 equipped with an RCS coolant system and Tzero aluminium hermetic pans and lids, using a heating rate of 10 ℃/min and a cooling rate of 5 ℃/min in the range of -70-150 ℃. The data was collected and analysed using TA software. The second heating curves were recorded, and glass transition temperatures of polymers were analysed.

**X-ray Photoelectron Spectroscopy (XPS).** XPS was conducted using a Kratos Axis SUPRA XPS fitted with a monochromated Al Kα X-ray source. Binding energies were calibrated using C 1s peak at 284.8 eV.

**Gel permeation chromatography (GPC).** GPC (CHCl_3_) was performed using Viscotek TDA302 instrument equipped with tetra detectors (RI, UV, RALS/LALS, and viscometer). The system was equipped with two T6000M Org GPC/SEC columns. The eluent is CHCl_3_. Samples were run at 0.8 mL/min at 40 ℃. Polystyrene solutions were used for calibration between 100 and 10,000,000 g/mol. Molecular weight and dispersity (Đ) values of obtained polymers were determined by RI conventional calibration using Omnisec GPC/SEC software. All analysed samples were the soluble part of polymers, and prepared the day before the test in the concentration of 3 mg/mL and filtrated through a 0.45 μm nylon syringe filter.

**Solubility test:** The solubility of the SED 5–7.5–6.25 polymer was assessed in various solvents. A total of 60 mg of the polymer was dispersed in 10 mL of solvent and allowed to stand for 24 h, followed by separation of the soluble and insoluble fractions. The insoluble fraction was subsequently dried in a vacuum oven at 60 °C for 24 h and weighed.

**Tensile Test.** Tensile stress-strain curves of polymers were carried out using a universal testing machine (SHIMADZU EZTest) with crosshead rate of 15 mm/min. The testing temperature was fixed at 22 °C using air conditioner. Dog-bone samples were made from polymer films responding to ISO 527-2/5A. The experimental procedure of making polymer films is illustrated in the next section. The average values with error bar of all mechanical data were obtained after 3~5 times tests for every sample.

**Film making and reprocessing experiments using hot press.** The polymers were hot pressed into uniform films, and were reprocessed, using a Hydraulic Lamination Hot Press purchased from Zhengzhou CY Scientific Instrument CO., LTD. Polymer films with average thickness of 1.00 mm were obtained through hot pressing inside a square metal mould with prescribed thickness. For original samples, the polymers were directly hot pressed after curing in the oven without any further processing. For recycling experiments, all polymers were cut into pieces then were hot pressed into new films. In principle, higher crosslinking density of the polymer requires higher temperature and longer pressing time to get a complete film. The reprocessing procedure was optimised as follows: Generally, S-DGEBA was hot pressed under 10 MPa at 100 °C for 10 min, S-ESO-DGEBA was hot pressed under 10 MPa at 80 °C for 10 min. Then, after cooling down to room temperature and unloading the mould, polymer films with the same thickness were obtained.

**Adhesion Testing:** Stainless-steel, PVC, wood, and glass substrates (2*6 cm) were employed for the adhesion tests. The PVC, wood, and glass substrates had a thickness of 3 mm and stainless-steel had a thickness of 1 mm. The substrates were abraded with 150-grit sandpaper to achieve a uniform surface roughness, followed by thorough cleaning with anhydrous ethanol to remove surface contaminants. Polymer films were prepared via hot pressing at 80 °C. A single layer of the polymer film was then applied onto the substrate with a bonding area of 1 × 2 cm. The amount of polymer used for each sample was 25 ± 2 mg. Subsequently, two substrates were aligned and fixed using a mechanical clamp, and the assembled specimens were heated in an oven at 130 °C for 15 min, while the PVC specimens were heated at 100 °C for 30 min. After cooling to ambient temperature, lap-shear adhesion tests were performed using a Universal Testing Machine model (INSTRON -3369) tensile testing machine at a rate of 2 mm min^-1^. The adhesive strength was calculated by dividing the maximum load at failure by the overlap area. Each sample was tested three times to ensure reproducibility.

**Shape memory and permanent reshaping experiments**: Polymer A was cut into a rectangular film, which then undergo three cumulative permanent reshaping deformations and three cycles of reversible temporary shape memory behavior. For temporary reshaping, the film was reshaped at 50 °C for a few seconds and maintained its shape at room temperature (approx. 20 °C), followed by reversible recovery at 70 °C for a few seconds. For permanent reshaping, the film was reshaped at 80 °C for 15 minutes and maintained its shape at room temperature. The new shape cannot recover to the original shape due to the topological rearrangement of the film that occurs during permanent reshaping.

Synthesis Methods

Synthesis of PMO-S with catalyst.

Sulfur (0.2 g, 0.78 mmol) and PMO (0.2 g) were added to a 10 mL glass vial equipped with a magnetic stirrer bar. Then, the vial was heated at 140 ℃ on a hotplate directly with 200 rpm stirring for 10 min. After sulfur melt and a yellow liquid was formed, 3 wt.% (based on sulfur) NaOH was added and the reaction mixture was further stirred at 900 rpm until the stir bar stopped rotating. The stirring was then stopped, and the product was analyzed by NMR, FT-IR spectroscopy. The obtained black product was denoted as PMO-S.

Control reaction (PMO and S_8_ react without catalyst).

As a control group to examine the effect of catalyst, control group was prepared under the same synthesis conditions as PMO-S. Sulfur (0.2 g, 0.78 mmol) and PMO (0.2 g) were added to a 10 mL glass vial equipped with a magnetic stir bar. The vial was then heated at 140 °C on a hot plate with continuous stirring at 200 rpm for 10 min. After the sulfur melted and a yellow liquid was formed, the reaction mixture was further stirred at 200 rpm for 24 h. The reaction was subsequently stopped, and the mixture was cooled to room temperature, revealing obvious phase separation. The upper epoxy layer changed from clear and colorless to pale orange-yellow, while the bottom layer contained a large amount of unreacted solid sulfur. The product was finally analyzed by FT-IR spectroscopy.

Synthesis of Poly-PMO.

As a control group to examine the effect of epoxide group self-polymerization under base catalyst, Poly-PMO was prepared under the same synthesis conditions as PMO-S. PMO (0.2 g) were added to a 10 mL glass vial equipped with a magnetic stir bar. The vial was then heated at 140 °C on a hot plate with continuous stirring at 200 rpm for 10 min. And then add 5 wt.% NaOH, the reaction mixture was further stirred at 200 rpm for 24 h. Cooling down to room temperature, product was tested by NMR, MS and FT-IR.

Study the effect of catalyst type on reaction rate.

NaOH, KOH, LiOH, triethylamine, NMI, MTBD and Zn (DTC)_2_ were chosen to study the effect of catalyst type on the reaction rate. The catalyst content was set to 3 wt.% (based on sulfur), and the synthesis was carried out following the same procedure as that for PMO-S.

Study the effect of catalyst content on reaction rate.

NaOH was chosen as the catalyst to study the influence of catalyst loading on the reaction rate. Sulfur (0.2 g, 0.78 mmol) and PMO (0.2 g) were added to a 10 mL glass vial equipped with a magnetic stirrer bar. Then, the vial was heated at 140 ℃ on a hotplate directly with 200 rpm stirring for 10 min. After sulfur melt and a yellow liquid was formed, 1 wt.%, 2 wt.%, 3 wt.%, 4 wt.%, 5 wt.% (based on sulfur) NaOH was added and the reaction mixture was further stirred at 900 rpm until the stir bar stopped rotating. The stirring was then stopped, and the product was analyzed by FT-IR spectroscopy.

Study the effect of reaction temperature on reaction rate.

NaOH was selected as the catalyst to investigate the effect of reaction temperature on the reaction rate. Sulfur (0.2 g, 0.78 mmol) and PMO (0.2 g) were added to a 10 mL glass vial equipped with a magnetic stir bar. For reactions conducted at 140 °C, the vial was heated on a hot plate with stirring at 200 rpm for 10 min. After the sulfur melted and a yellow homogeneous liquid was formed, 3 wt.% NaOH (based on sulfur) was added, and the reaction mixture was further stirred at 900 rpm until the stir bar stopped rotating.

For reactions carried out at 100, 80, and 50 °C, as well as at room temperature (RT), 3 wt.% NaOH (based on sulfur) was added, and the reaction mixture was stirred at 200 rpm for different durations. The mixture was allowed to cool to RT, and the resulting product was analyzed by FT-IR spectroscopy.

Study the effect of adventitious moisture.

In order to evaluate the influence of adventitous water on the reaction, all solid reagents and glassware were handled in an Ar-filled glovebox. Sulfur was dried prior to use, and sodium hydroxide was ground into a fine powder before being transferred into the glovebox. A dried reaction flask equipped with a dried magnetic stir bar, together with the nitrogen-purged monomer, was also transferred into the glovebox. The monomer was used as received from a commercial supplier and was not subjected to rigorous drying.

The required amounts of sulfur, sodium hydroxide, and monomer were weighed inside the glovebox and added to the dried reaction flask. The flask was sealed with a rubber septum under the Ar atmosphere and then taken out of the glovebox. The sealed flask was heated at 140 °C with stirring. After completion of the reaction, the resulting product was analyzed by NMR spectroscopy and ESI-MS.

Synthesis of OLO-S.

OLO-S was synthesized following the same procedure as PMO-S. Sulfur (0.2 g, 0.78 mmol) and OLO (0.2 g) were added to a 10 mL glass vial equipped with a magnetic stirrer bar. Then, the vial was heated at 140 ℃ on a hotplate directly with 200 rpm stirring for 10 min. After sulfur melt and a yellow liquid was formed, 5 wt.% (based on sulfur) NaOH was added and the reaction mixture was further stirred at 900 rpm until the stir bar stopped rotating. The stirring was then stopped, and the product was analyzed by NMR, MS and FT-IR spectroscopy. The obtained black product was denoted as OLO-S.

Synthesis of Poly-OLO.

As a control group to examine the effect of epoxide group self-polymerization under base catalyst, Poly-PMO was prepared under the same synthesis conditions as OLO-S. OLO (0.2 g) were added to a 10 mL glass vial equipped with a magnetic stir bar. The vial was then heated at 140 °C on a hot plate with continuous stirring at 200 rpm for 10 min. And then add 5 wt.% NaOH, the reaction mixture was further stirred at 200 rpm for 24 h. Cooling down to room temperature, product was tested by NMR, MS and FT-IR. The obtained yellow product was denoted as OLO-S.

Synthesis of DGEBA-S.

The polymer DGEBA-S was synthesized as follows. Sulfur (11.26 g, X mmol) and DGEBA (7.5 g, X mmol) (epoxy-to-sulfur molar ratio of 1:8) were added to a 40 mL glass vial equipped with a magnetic stirrer bar. Then, the vial was heated at 140 ℃ on a hotplate directly with 200 rpm stirring for 10 min. After sulfur melt and a yellow liquid was formed, 3 wt.% (based on sulfur) NaOH was added and the reaction mixture was further stirred at 900 rpm for 8 min until the stir bar stopped rotating. And keep the temperature at 140 ℃ for curing overnight. The product was analyzed by NMR, MS and FT-IR spectroscopy. The obtained black product was denoted as DGEBA-S.

Synthesis of ESO-S.

The polymer ESO-S was synthesized as follows. Sulfur (5 g) and ESO (5 g) were added to a 40 mL glass vial equipped with a magnetic stirrer bar. Then, the vial was heated at 140 ℃ on a hotplate directly with 200 rpm stirring for 10 min. After sulfur melt and a yellow liquid was formed, 3 wt.% (based on sulfur) NaOH was added and the reaction mixture was further stirred at 900 rpm for 60 min until the stir bar stopped rotating. And keep the temperature at 140 ℃ for curing overnight. The product was analysed by NMR, MS and FT-IR spectroscopy. The obtained black product was denoted as ESO-S.

Synthesis of terpolymer SED.

The terpolymer SED was synthesized as follows. Sulfur and ESO were added to a 40 mL glass vial equipped with a magnetic stir bar. The vial was then heated at 140 °C on a hot plate with stirring at 200 rpm for 10 min. After the sulfur melted and a yellow liquid was formed, 5 wt.% NaOH (based on sulfur) was added, and the reaction mixture was further stirred at 900 rpm for 60 min. At this temperature, preheated DGEBA was added to the vial, and the mixture was maintained at 140 °C with continuous stirring for an additional 10 min until the stir bar stopped stirring. The reaction mixture was then cured at 140 °C overnight. The resulting product was characterized by NMR, MS, and FT-IR spectroscopy. The obtained black product was denoted as SED.

Figure S1. Reaction progress of PMO-S. a, S_8_ and PMO melting at 140 °C. b, S_8_ and PMO reacted at 140 °C without NaOH after 4 and 24 hours, respectively. c, S_8_ and PMO reacted at 140 °C with 3 wt.% NaOH after 10min. d, PMO reacted at 140 °C with NaOH after 24h.

Figure S2. The ^1^H liquid-state NMR spectra of PMO and PMO reacted with S_8_ without NaOH.

 Figure S3. The ^1^H NMR and ^13^C liquid-state NMR spectra of PMO and PMO-S in CDCl_3_.

Figure S4. The DEPT-135 NMR spectra of PMO-S in CDCl_3_.

Figure S5. The H-H NMR spectra of PMO-S in CDCl_3_.

Figure S6. The H-C NMR spectra of PMO-S in CDCl_3_.

Figure S7. MS spectra of PMO-S obtained at 140 °C in the presence of NaOH.

Figure S8. The ^1^H liquid-state NMR spectra of Poly-PMO in CDCl_3_.

Figure S9. The ^13^C liquid-state NMR spectra of Poly-PMO in CDCl_3_.

Figure S10. The DEPT-135 liquid-state NMR spectra of Poly-PMO in CDCl_3_.

Figure S11. MS spectra of Poly-PMO obtained at 140 °C in the presence of NaOH.

Figure S12. FT-IR spectra of Poly-PMO and PMO-S obtained at 140 °C in the presence of NaOH.

Figure S13. Comparison of PMO react at different conditions.

Figure S14. Images of PMO-S samples prepared using different catalysts (140 °C, 3 wt% catalyst).

Table S1: A summary of the catalytic effects of various catalysts.

|  | **Reactivity** | **Min reaction time (at 140 °C)** | **Min reaction Temperature** |
| --- | --- | --- | --- |
| Blank | × | × | × |
| NaOH | √ | ＜10 min | 50 °C (＞2 days) |
| Triethylamine | √ | ＞8 h | - |
| N-methylimidazole (NMI) | √ | ＞8 h | - |
| Zn (DTC)_2_ | √ | ＞8 h | - |
| MTBD | √ | ＜1 min | RT (＞2 days) |
| KOH | √ | > 8 h | 100 °C (> 24 h) |
| LiOH | √ | ＜60 min | 100 °C (＞12h) days) |

Table S2. Effect of catalyst loading on the reaction efficiency.

| **NaOH content** | **Reaction rate** |
| --- | --- |
| 1% | Slow, after 4h can be stirred |
| 2% | Slow, after 2h can be stirred |
| 3% | Quick, 10min solid, can not be stirred |
| 4% | Very quick, 5min solid, can not be stirred |
| 5% | Very quick, 5min solid, can not be stirred |

Figure S15. Images of PMO-S obtained by a, NaOH b, KOH, c, LiOH at 100 °C for different reaction times.

Figure S16. FT-IR spectra PMO-S catalyzed by NaOH at 100 °C for different reaction times.

Figure S17. Images of PMO-S obtained by NaOH at 80 °C for different reaction times.

Figure S18. FT-IR spectra PMO-S catalyzed by NaOH at 80 °C for different reaction times.

Figure S19. Images of PMO-S obtained by NaOH at 50 °C for different reaction times.

Figure S20. FT-IR spectra PMO-S catalyzed by NaOH at 50 °C for different reaction times.

Figure S21. MS spectra of PMO-S obtained at 50 °C in the presence of NaOH.

Table S3: Effect of reaction temperature on the reaction efficiency (NaOH).

| **Reaction temperature** | **Reaction rate** |
| --- | --- |
| 140 | Very quick, 10 min solid, can not be stirred |
| 100 | Quick, ＜8h |
| 80 | Slow, ＞8h |
| 50 | Slow, ＞8h |
| RT | Non-reactive |

Figure S22. Images and FT-IR spectra PMO-S catalysed by MTBD at 140 °C for different reaction times.

Figure S23. Images of PMO-S catalysed by MTBD at room temperature for different reaction times.

Figure S24. FT-IR spectra PMO-S catalysed by MTBD at room temperature for different reaction times.

**Mechanism study:**

Figure S25. a, Images of the reaction between PMO and S_8_ in the presence of TEMPO (TEMPO was added first, followed by NaOH). b, Images after 1 h of reaction.

Figure S26. a, ^1^H-NMR spectra of PMO-S obtained in the presence of TEMPO.

Figure S27. Reaction progress of PMO-S. a, Na_2_S_5_. b, Na_2_S_5_ and PMO melting at 140 °C. c, Na_2_S_5_ and PMO reacted at 140 °C after 72h.

Figure S28. ^1^H-NMR spectra of PMO-S obtained from Na_2_S_5_ react with PMO.

Figure S29. ESI-MS of PMO-S from Na_2_S_5_ react with PMO.

Figure S30. FT-IR of PMO-S from Na_2_S_5_.

Figure S31. Images of the reaction between PMO and S_8_ under Ar. b, Images after 10 min of reaction. c, No obvious gas was generated after the reaction. d, Comparison images of the reactions under Ar (left) and air condition (right) after 10 min.

Figure S32. a, ^1^H NMR, b, ^13^C NMR, c, DEPT of PMO-S obtained under Ar.

Figure S33. ESI-MS of PMO-S obtained under Ar.

Figure S34. MS spectra of PMO-S obtained at 50 °C in the presence of NaOH.

Figure S35. Reaction progress of OLO-S. a, S_8_ and OLO melting at 140 °C. b, S_8_ and OLO reacted at 140 °C with NaOH after 30 min. c, OLO reacted at 140 °C with NaOH in the absence of S_8_ after 10min and 24h, respectively.

Figure S36. The ^1^H liquid-state NMR spectra of OLO and OLO-S in CDCl_3_.

Figure S37. The ^13^C liquid-state NMR spectra of OLO and OLO-S in CDCl_3_.

Figure S38. The DEPT-135 liquid-state NMR spectra of OLO-S in CDCl_3_.

Figure S39. The ^1^H-NMR liquid-state NMR spectra of Poly-OLO compare with OLO and OLO-S in CHCl_3_.

Figure S40. The ^1^H liquid-state NMR spectra of Poly-OLO in CDCl_3_.

Figure S41. The ^13^C liquid-state NMR spectra of Poly-OLO in CDCl_3_.

Figure S42. The DEPT-135 liquid-state NMR spectra of Poly-OLO in CDCl_3_.

Figure S43. FT-IR spectra OLO, Poly-OLO and OLO-S.

| **2OLO + 2H + Na + nS** |  | **2S** | **3S** | **4S** | **5S** | **6S** | **7S** |
| --- | --- | --- | --- | --- | --- | --- | --- |
|  | **Theoretical** | 345.1892 | 377.1613 | 409.1334 | 441.1054 | 473.0775 | 505.0496 |
|  | **Found** | 345.1824 | 377.1605 | 409.0986 | 441.0722 | 473.0421 | 505.0996 |

Figure S44. ESI-MS of OLO-S from S_8_ react with OLO under NaOH.

Figure S45. The ESI-MS of Poly-OLO.

Figure S46. Reaction progress of DGEBA-S. a, S_8_ and DGEBA melting at 140 °C. b, S_8_ and DGEBA reacted at 140 °C without NaOH after 24 h. c, S_8_ and DGEBA reacted at 140 °C with NaOH after 5 min. d, DGEBA reacted at 140 °C with NaOH after 24h.

Figure S47. The ^1^H NMR liquid-state NMR spectra of DGEBA and DGEBA-S in CDCl_3_.

C-O

Figure S48. FT-IR plot of DGEBA-S and Poly-DGEBA.

Figure S49. DSC plot of DGEBA-S and Poly-DGEBA.

Figure S50. a, DGEBA-S in CHCl_3_; b, soluble part, c, insoluble part.

Table S4. CHNS. (soluble fraction 52.9%, insoluble fraction 47.1%)

| **polymer** | **-** | **C** | **H** | **S** | **O & Na** | **C/H** |
| --- | --- | --- | --- | --- | --- | --- |
| DGEBA-S 50:50 | theoretical | **36.53** | **3.54** | **49.26** | **10.7** | **10.42** |
|  | experimental | **36.09** | **3.36** | **49.96** | **10.59** | **10.74** |
| soluble fractions | experimental | **27.14** | **2.72** | **64.74** | **5.4** | **9.98** |
| insoluble fractions | experimental | **47.28** | **4.53** | **26.24** | **21.95** | **10.44** |

Figure S51. a. Soluble and b. insoluble fractions of DGEBA-S.

Figure S52. Reaction progress of ESO-S. a, S_8_ and ESO melting at 140 °C. b, S_8_ and ESO reacted at 140 °C without NaOH after 24 h. c, S_8_ and ESO reacted at 140 °C with NaOH after 24h. d, ESO reacted at 140 °C with NaOH after 24h.

Figure S53. ^1^H-NMR of ESO in CDCl_3_.

Figure S54. The ^1^H NMR liquid-state NMR spectra of ESO, poly-ESO and ESO-S in CDCl_3_.

Figure S55. The FT-IR plot of ESO and ESO-S.

Figure S56. The DSC curves of Poly-ESO and ESO-S.

Table S5: Molecular weight parameters of poly-ESO, ESO-S, and DGEBA-S determined by GPC in CHCl_3_.

|  | **Mn (g/mol)** | **Mw (g/mol)** | **PD** |
| --- | --- | --- | --- |
| Poly-ESO | 2224 | 2226 | 1.000931 |
| ESO-S | 159078 | 434897 | 2.733859 |
| DGEBA-S* | 68903 | 81104 | 1.177086 |

*: DGEBA-S for soluble portion.

Table S6: Summary of SED terpolymers with different weight ratios.

|  | **Sulfur / g** | **ESO / g** | **DGEBA / g** | **S content wt.%** |
| --- | --- | --- | --- | --- |
| 5-5-x | 5 | 5 | 0.83 | 0.46 |
|  | 5 | 5 | 1.67 | 0.43 |
|  | 5 | 5 | 2.5 | 0.40 |
|  | 5 | 5 | 3.33 | 0.38 |
|  | 5 | 5 | 4.17 | 0.35 |
|  | 5 | 5 | 5 | 0.33 |
| 5-7.5-x | 5 | 7.5 | 3.75 | 0.31 |
|  | 5 | 7.5 | 5 | 0.29 |
|  | 5 | 7.5 | 6.25 | 0.27 |
|  | 5 | 7.5 | 7.5 | 0.25 |
|  | 5 | 7.5 | 10 | 0.22 |
| 5-10-x | 5 | 10 | 5 | 0.25 |
|  | 5 | 10 | 6.77 | 0.23 |
|  | 5 | 10 | 8.33 | 0.21 |
|  | 5 | 10 | 10 | 0.20 |

Figure S57. XPS of DGEBA-S.

Figure S58. XPS of ESO-S.

Figure S59. XPS of SED-5-7.5-6.25.

Figure S60: Solubility test of the SED terpolymer 5-7.5-6.25 in different solvents.

Table S7: Gel fraction of SED 5-7.5-6.25 in different solvents.

| Solvent | CH_2_Cl_2_ | CHCl_3_ | EtOH | THF | DMF | DMSO | Acetone |
| --- | --- | --- | --- | --- | --- | --- | --- |
| Gel fraction / % | 60.83 | 63.44 | 87.17 | 54.80 | - | - | 55.27 |

Figure S61: DSC curves of SED terpolymer series 5-5-x.

Figure S62: DSC curves of SED terpolymer series 5-7.5-x.

Figure S63: DSC curves of SED terpolymer series 5-10-x.

Figure S64: TGA curves of SED terpolymers.

Figure S65: Images of a. polymer SED-5-7.5-6.25, b. Dog-bonds of polymer SED-5-7.5-6.25; c-d. stretching processes.

Figure S66. The typical strain-stress curves of SDE-5-5-X sulfur-polymers

Figure S67. The typical strain-stress curves of SDE-5-10-X sulfur-polymers.

Figure S68. Typical stress–strain curves of the SDE-5-7.5-6.25 sulfur polymer during recycling cycles.

Figure S69. The DSC plot of SDE-5-7.5-6.25 before and after three times tensile testing.

Figure S70. Lap shear curves of SED-5-5-X adhesive on steel sheets at room temperature (20 °C).

Figure S71. lap shear strength of different SED cross-linked polymers (5-5-x) with stain-less steel sheet.

Figure S72. Lap shear curves of SED-5-7.5-X adhesive on steel sheets at room temperature (20 °C).

Figure S73. Reusability test. Lap shear curves of SED-5-7.5-6.25 bonded steel sheets at room temperature (20 °C).

Figure S74. Reusability test. Lap shear strength of SED 5-7.5-6.25 cross-linked polymers with stain-less steel sheet. (20 °C).

Figure S75. Lap shear curves of commercial epoxy AB glue bonded steel sheets at room temperature (20 °C). Lap shear stress is 11.1±1.78 MPa.

Figure S76. Lap shear curves of SED-5-7.5-6.25 adhesive on wood at room temperature (25 °C).

Figure S77. Lap shear curves of SED-5-7.5-6.25 adhesive on PVC at room temperature (25 °C).

Figure S78. Lap shear curves of SED-5-7.5-6.25 adhesive on glass at room temperature (25 °C).

Figure S79: lap shear strength of SED 5-7.5-6.25 cross-linked polymers with different substract.

Table S8: Lap shear strength of the typical polysulfide adhesive examples.

| Adhesives | Adhesion strength (MPa) | | | | | Methods | References |
| --- | --- | --- | --- | --- | --- | --- | --- |
|  | Fe | Stain-less steel | Al | Wood | Glass |  |  |
|  | - | 25 | - | - | - | Dry: kept in a 190 °C oven for 2 hours | Yu Jin ^[74]^ |
|  | - | 32 | - | - | - | Wet: cured *in situ* in a 160 °C oven for 4 hours |  |
|  | - | - | - | - | - | Heating at 140 °C for 10 min | Zhongkai Guo ^[75]^ |
|  | 7 | - | 4 | 12 | 8 | - | Yi Wang ^[76]^ |
|  | - | 0.03 | - | - | - | - | Seong-Ju Lee ^[77]^ |
|  | - | 11 | 7 | 9.6 | 0.8 | 15 minutes of hot pressing at 130 °C | Jieai Fan ^[78]^ |
|  | - | 2.23 | 2.08 | 1.09 | - | Pressing at room temperature for 2–5 minutes | Huishan Huang ^[79]^ |
|  | - | 7.3 | 5.8 | - | - | Curing in an oven at 110 °C for 24 h | Shuojia Zheng ^[80]^ |
|  | 1.91 | - | 1.88 | 3.2 | 1.0 | Cold pressing at room temperature for 3 min | Qingyong Chen ^[81]^ |
|  | - | - | - | - | - | 4 cm^2^ polymer pieces were attached, using pyridine or triethylamine to assist adhesion for 24 hours | Samuel J. Tonkin ^[82]^ |
|  | - | 2.8 | - | 2.5 | - | placed in the oven at 60 °C to complete curing | Hongzhen Liu ^[83]^ |
|  | - | - | 3.2 | - | - | Heated at 120 °C for 30 min | Hang Shen ^[84]^ |
|  | - | - | 2.7 | - | - | Cured at 160 °C for 24 h | Kyler B. Sayer ^[85]^ |
|  | - | 1.3 | - | 0.8 | 0.5 | Get liquid copolymers at 140 °C, add dropwise to the surface of substrates | Jinhong Jia ^[86]^ |
|  | - | 10 | 12 | - | 7.5 | The polymers were molten by heating at 120 °C and then deposited on the surfaces of substrates | Yuanxin Dong ^[87]^ |
| Our work | - | 10 | - | 5.9 | >4.2 | Heated at 130 °C for 15 min | Our work |

**References**

[76]	Y. Jin, Z. Wang, C. Hu, J. Wang, K. Yan, J. He, Z. Wang, Z. Wang, L. Yuan, “Chemically debondable, high-strength and tough adhesives from sulfur-modified epoxy networks” Green Chemistry 2023, 25, 1157–1168.

[77]	Z. Guo, X. Jiao, K. Wei, J. Wu, J. Hu, “Comprehensive itaconic acid-based vitrimers via one-pot inverse vulcanization” Green Chemistry 2023, 25, 4544–4552.

[78]	Y. Wang, F. Zhang, F. Li, S. Du, S. Dai, T. Jiang, K. Sun, B. Chen, S. Ma, “Covalent adaptable networks derived from itaconic acid-based epoxy and sulfur: Facile synthesis and applications in adhesives and carbon fiber composites” Composites Communications 2025, 59, 102575.

[79]	S.-J. Lee, G.-Y. Han, M.-B. Yi, J.-H. Back, H.-J. Kim, “From waste to tape: Inverse vulcanization of sulfur and solvent-based depolymerization for preparation of pressure-sensitive adhesives” Journal of Materials Research and Technology 2024, 29, 1798–1804.

[80]	J. Fan, C. Ju, S. Fan, X. Li, Z. Zhang, N. Hadjichristidis, “Inverse Vulcanization of Aziridines: Enhancing Polysulfides for Superior Mechanical Strength and Adhesive Performance” Angewandte Chemie International Edition 2025, 64, e202418764.

[81]	H. Huang, S. Zheng, J. Luo, L. Gao, Y. Fang, Z. Zhang, J. Dong, N. Hadjichristidis, “Step-growth Polymerization of Aziridines with Elemental Sulfur: Easy Access to Linear Polysulfides and Their Use as Recyclable Adhesives” Angewandte Chemie International Edition 2024, 63, e202318919.

[82]	S. Zheng, Y. Deng, J. Yao, C. Ju, W. Yang, P. Gao, W. Lai, J. Luo, Z. Zhang, “Intrinsically Flame-Retardant and Recyclable Adhesives from N-Phosphorylated Aziridine-Based Inverse Vulcanized Polymers” Industrial & Engineering Chemistry Research 2025, 64, 20876–20885.

[83]	Q. Chen, L. Xu, L. Zhu, L. Gao, J. Luo, X. Yang, Y. Fang, Z. Zhang, J. Dong, “Aziridine-Derived Polysulfide Elastomers for Self-Healable, Strong, and Reusable Adhesives” ACS Applied Polymer Materials 2024, 6, 6437–6447.

[84]	S. J. Tonkin, C. T. Gibson, J. A. Campbell, D. A. Lewis, A. Karton, T. Hasell, J. M. Chalker, “Chemically induced repair, adhesion, and recycling of polymers made by inverse vulcanization” Chemical Science 2020, 11, 5537–5546.

[85]	H. Liu, S. Cao, Z. Liu, J. Wang, Y. Liu, L. Sun, C. Gao, “Sulfur-modified polyurethane adhesives: Green synthesis process and disassembly-responsive characteristics” Polymer Degradation and Stability 2024, 228, 110910.

[86]	H. Shen, H. Qiao, H. Zhang, “Sulfur-urushiol copolymer: A material synthesized through inverse vulcanization from renewable resources and its latent application as self-repairable and antimicrobial adhesive” Chemical Engineering Journal 2022, 450, 137905.

[87]	K. B. Sayer, V. L. Miller, Z. Merrill, A. E. Davis, C. L. Jenkins, “Allyl sulfides in garlic oil initiate the formation of renewable adhesives” Polymer Chemistry 2023, 14, 3091–3098.

[88]	J. Jia, Y. Chai, X. Xun, Y. Gao, T. Qiao, X. Wang, X.-C. Wang, T. Hasell, X. Wu, Z.-J. Quan, “Dynamic Covalent Sulfur-Selenium Rich Polymers via Inverse Vulcanization for High Refractive Index, High Transmittance, and UV Shielding Materials” Macromolecular Rapid Communications 2025, 46, 2400998.

[89]	Y. Deng, Z. Huang, B. L. Feringa, H. Tian, Q. Zhang, D.-H. Qu, “Converting inorganic sulfur into degradable thermoplastics and adhesives by copolymerization with cyclic disulfides” Nature Communications 2024, 15, 3855.
